# Statin Therapy in Post-Operative Atrial Fibrillation: Focus on the Anti-Inflammatory Effects

**DOI:** 10.3390/jcdd8030024

**Published:** 2021-02-26

**Authors:** Homa Nomani, Amir Hooshang Mohammadpour, Željko Reiner, Tannaz Jamialahmadi, Amirhossein Sahebkar

**Affiliations:** 1 School of Pharmacy, Mashhad University of Medical Sciences, Mashhad 9179156314, Iran; Nomanih8@mums.ac.ir; 2 Pharmaceutical Research Center, Pharmaceutical Technology Institute, Mashhad University of Medical Sciences, Mashhad 9179156314, Iran; Mohamadpoorah@mums.ac.ir; 3 Department of Clinical Pharmacy, School of Pharmacy, Mashhad University of Medical Sciences, Mashhad 9179156314, Iran; 4 Department of Internal Medicine, University Hospital Ceter Zagreb, School of Medicine University of Zagreb, 10000 Zagreb, Croatia; zreiner@kbc-zagreb.hr; 5 Department of Food Science and Technology, Quchan Branch, Islamic Azad University, Quchan 9479176135, Iran; jamiat981@mums.ac.ir; 6 Department of Nutrition, Mashhad University of Medical Sciences, Mashhad 9177948564, Iran; 7 Biotechnology Research Center, Pharmaceutical Technology Institute, Mashhad University of Medical Sciences, Mashhad 9177948564, Iran; 8 Applied Biomedical Research Center, Mashhad University of Medical Sciences, Mashhad 9177948564, Iran; 9 Polish Mother’s Memorial Hospital Research Institute (PMMHRI), 93-338 Lodz, Poland

**Keywords:** atrial fibrillation, inflammation, cardiac surgery, statins, atorvastatin, rosuvastatin

## Abstract

Background: Atrial fibrillation (AF) occurring after cardiac surgery, post-operative AF (POAF), is a serious and common complication of this treatment. POAF may be life-threatening and the available preventive strategies are insufficient or are associated with significantly increased risk of adverse effects, especially in long-term use. Therefore, more appropriate treatment strategies are needed. Methods: In this paper, the efficacy, safety, and other aspects of using statins in the prevention of POAF focusing on their anti-inflammatory effects are reviewed. Results: Recent studies have suggested that inflammation has a significant role in POAF, from the first AF episode to its serious complications including stroke and peripheral embolism. On the other hand, statins, the most widely used medications in cardiovascular patients, have pleiotropic effects, including anti-inflammatory properties. Therefore, they may potentially be effective in POAF prevention. Statins, especially atorvastatin, appear to be an effective option for primary prevention of POAF, especially in patients who had coronary artery bypass grafting (CABG), a cardiac surgery treatment associated with inflammation in the heart muscle. However, several large studies, particularly with rosuvastatin, did not confirm the beneficial effect of statins on POAF. One large clinical trial reported higher risk of acute kidney injury (AKI) following high-dose rosuvastatin in Chinese population. In this study, rosuvastatin reduced the level of C-reactive protein (CRP) but did not reduce the rate of POAF. Conclusion: Further studies are required to find the most effective statin regimen for POAF prevention with the least safety concern and the highest health benefits.

## 1. Introduction

The occurrence of atrial fibrillation (AF) after cardiac surgery, post-operative AF (POAF), is a serious and common post-operative complication [1]. POAF may be life-threatening [2] and the available preventive strategies are insufficient or are associated with significantly increased risk of adverse effects, especially in long-lasting treatment [3]. Therefore, finding more appropriate strategies is a concern. Recent studies have suggested that inflammation has a significant role in POAF, from the first episode of AF to serious complications including strokes and peripheral embolism [4]. Statins are the most widely used medications in cardiovascular patients and have many pleiotropic effects apart from their established low-density lipoprotein (LDL)-lowering activity [5,6,7,8,9,10,11,12]. In this paper, a summary of the role of inflammation in the POAF is presented as well as the evidence indicating anti-inflammatory effects of statins. The recent clinical evidence on the effects of statins on POAF are also extensively reviewed.

## 2. The Role of Inflammation in AF

Several epidemiologic studies confirmed the association between AF and inflammation as indicated by the elevation of inflammatory biomarkers like C-reactive protein (CRP) [13]. The peak CRP level occurs on the second post-operative day after cardiac surgery, and POAF typically occurs within the first three post-operative days [13]. However, the important question is how does surgery trigger the inflammation and how could this result in POAF. During cardiac surgery, inflammation could be induced by factors like surgical injury, myocardial ischemia and a hyper-adrenergic state [14]. Inflammation causes the production of inflammatory mediators: cytokines.

These markers also can be secreted by epicardial adipose tissue (EAT) and may have local pro-inflammatory effects on the atrial myocardium, which might cause cardiac arrhythmia [15].

Epicardial adipose tissue (EAT) is located along the myocardium and the visceral layer of the pericardium. It is considered to be an endocrine organ that releases pro-inflammatory cytokines associated with arrhythmia. Epicardial fat directly or indirectly promotes atrial fibrillation (AF) by atrial remodeling. It can penetrate the atrium and alter its electrophysiological structure (direct pathway) or modulate cardiac inflammation and oxidative stress in a paracrine fashion (indirect pathway). Additionally, the interaction between EAT and pulmonary veins is likely to have an arrhythmogenic effect and prevent AF [15,16].

Cytokines can trigger changes in the atrium, i.e., electrical and structural remodeling of the atrium by different mechanisms [17]. These changes cause conduction heterogeneity and are predisposition for AF [18]. The mechanisms by which inflammation can cause the atrium remodeling and POAF include oxidative stress, myolysis, apoptosis, and reduction of connexin 40 and 43 [19], the gap junction proteins expressed in the atrium and involved in normal atrial conduction [20]. Abnormal calcium handling is another important mechanism that results in abnormal conduction and conduction heterogeneity [19]. Inflammation can trigger POAF, especially after coronary artery bypass grafting (CABG), as an intensive and aggressive cardiac surgery [21]. Therefore, drugs with anti-inflammatory properties may be potentially effective in primary prevention of POAF.

### Drugs with Anti-Inflammatory Properties and POAF

The effectiveness of medical interventions to prevent or reduce the occurrence of POAF has been previously evaluated. Several anti-inflammatory agents such as nonsteroid anti-inflammatory drugs (NSAIDs), steroid anti-inflammatory drugs, immunosuppressants, and statins can prevent POAF. In addition, colchicine, a drug used for gout management, can be safe and useful for peri-operative inhibition of inflammation and may also be effective in the prevention of POAF [22].

## 3. Anti-Inflammatory Effects of Statins

Statins are inhibitors of 3-hydroxy-3-methylglutaryl coenzyme A (HMG-CoA) reductase. They are designed to decrease blood cholesterol, particularly LDL-cholesterol [23,24]. However, already more than three decades ago, it was shown that they have several other beneficial effects, i.e., pleiotropic effects, independent from their cholesterol-lowering properties [23]. There are several mechanisms by which statins might be involved in preventing AF including the improvement of endothelial function, their antioxidative, and anti-inflammatory effects [13]. The focus of this paper is on anti-inflammatory effects of statins.

There are many in vitro and animal studies suggesting that statins have considerable anti-inflammatory properties [23]. Recent clinical studies suggest that this effect is beneficial and is independent of their lipid-lowering effect [23].

The most convincing clinical evidence indicating the anti-inflammatory effects of statins is provided by studies analyzing the blood levels of inflammatory biomarkers such as CRP [23]. Several large clinical trials indicated that statin therapy could significantly reduce CRP level compared with placebo [25,26,27]. Statins also reduce the level of pro-inflammatory cytokines such as tumor necrosis factor-α (TNF-α), interleukin-1 (IL-1), and interleukin-6 (IL-6) [13].

Statins improve cardiometabolic status in patients with cardiovascular risk factors or overt cardiovascular diseases. Long-term statin therapy reduces EAT in patients with coronary artery disease. However, the role of short-time statin treatment on EAT volume in AF patients is still unclear [16].

Based on recent studies, the mechanisms by which statins exert their anti-inflammatory properties include reducing the number of inflammatory cells and cytokines, inhibiting the adhesion molecules, upregulation of the endothelial nitric oxide (NO), and inhibition of superoxide release [13].

## 4. Statins in the Primary Prevention of POAF: The Review of Available Clinical Evidence

There are several randomized clinical trials (RCTs) (Table 1) and meta-analyses (Table 2) that evaluated the effect of statin therapy on prevention of POAF. Some of these studies analyzed the changes in inflammatory markers to evaluate whether this effect is associated with anti-inflammatory effects of statins. Based on these studies, different aspects of statin therapy for primary prevention of POAF are discussed below.

### 4.1. Statin Effectiveness

Several RCTs [26,27,28,29,30,31,32,33,34,36] and meta-analyses [37,38,39,40,42,43,44,45,46,47,48,49] have shown that pre- or peri-operative statin therapy is effective in lowering the risk of POAF incidence with a range between 30% [49] and 59% [46] compared with placebo, non-statin, or low-dose statins, regardless of statin type, dose, and duration or surgery type. Statin therapy reduced the levels of inflammatory markers, especially CRP levels (Table 1 and Table 2) [50]. However, there are several limitations to the current data regarding statins for preventing AF, which are discussed below. As well, it is necessary to discuss different aspects of optimal statin therapy for POAF prevention and such a comprehensive discussion on drug selection, dose response, effectiveness associated to surgery type, and safety considerations will follow.

As we noted before, there are several limitations to the current data regarding statins for AF prevention, discussed here. First, a proportion of the current data are subgroup or post hoc analyses of randomized trials, of which some of them were not designed for the development of AF as a primary end point. Second, significant heterogeneity exists between and within studies on the method and the length of AF diagnosis or monitoring, which could significantly affect the rate of reported or diagnosed AF. Another important source of heterogeneity between studies may be the AF history of the study population, since primary prevention from AF is different from secondary prevention. Furthermore, patients who experienced AF are more prone to POAF occurrence [51]. Third, the type and the dose of statins were not systematically reported. A wide range of therapeutic potency, dosing regimens, and ethnic-based efficacy within this class of medications introduces potential statistical confounders. Finally, there are often contradictory results among meta-analyses, randomized, and observational studies, which confound any conclusions drawn [14]. There are some studies that showed no significant relation between statin therapy and POAF risk reduction. However, most of these studies were observational studies [52] and their results are not as reliable as experimental data because of more risk of bias or more detection of preexisting or asymptomatic AF. In addition, there were few clinical trials and just one meta-analysis failed to find statin therapy useful in POAF reduction. The most important trial with negative results was the STICS (statin therapy in cardiac surgery) trial. This trial was the largest, well-designed study, which used high-dose rosuvastatin in Chinese patients and led to significantly more post-operative acute kidney injury (AKI) [25]. The negative results of this study are discussed later. Interestingly, the only one meta-analysis, which failed to find statin therapy useful in reducing POAF rate [41], was greatly influenced by the STICS trial. However, two most recent meta-analyses results, which included this large trial, remained positive. These recent meta-analyses showed that statins were able to decrease the risk of POAF by 30–50% [37,38]. However, these meta-analyses confirmed the effectiveness of statins only in post-CABG patients [37,38]. Other trials had some limitations, which might affect their results. In the Billings et al. study, POAF was not an end point. As well, the majority of the population in this placebo-controlled study were statin users by the time of randomization. These patients resumed their pre-study statin regimen on post-operative day 2 [29], regardless of their study group, placebo, or statin group. Such a study design could greatly influence their results. In addition, they initiated statin therapy from the day before surgery, while almost all of the studies with POAF end point initiated statin therapy from a few days before surgery. Another two studies that failed to find statin therapy useful in POAF reduction did not compare statins with placebo or usual care. They compared high-dose atorvastatin with medium or low dose of that. Indeed, the results of these dose-comparing studies cannot evaluate statin efficacy.

Overall, statin therapy appeared to be an effective option for primary prevention of POAF [48], especially in CABG patients.

As previous studies indicated, there is a significant reduction in the probability of atrial fibrillation following open heart surgery with statin therapy. It is suggested that peri-operative statin treatment has no adverse side effects and its beneficial implications preponderate the potential risks. Also, according to the available guidelines, pre-operative statin therapy and post-operative re-initiation appear to be reasonable in high-risk patients [53].

### 4.2. Drug Selection and Effectiveness

Most of the studies confirming statins’ efficacy in reducing POAF risk were carried out with atorvastatin [27,30,31,32,33,34,36]. However, there are studies using other statins such as rosuvastatin [26], simvastatin [38], or pravastatin [38,54] but the results on the efficacy of these statins are conflicting. These results could be explained by different properties among different statins, especially pleiotropic effects such as anti-inflammatory properties [4]. However, the number of studies focusing on a specific statin or comparing their effects on AF prevention is still insufficient, and this fact might affect the results.

Two recent meta-analyses of RCTs found a significant association between statin therapy and decreased risk of POAF, particularly in the atorvastatin users [38,42]. These studies failed to show any association between POAF risk reduction and rosuvastatin [38,42] or simvastatin [38]. The largest RCT, which rejected rosuvastatin efficacy in POAF reduction, was the STICS trial [25]. This trial was conducted on a Chinese population and resulted in these conflicting findings. There are some explanations for these results. One possible explanation regards the race of the patients, who were Chinese and received as high as 20 mg daily rosuvastatin or placebo. According to a previous study, lower dosages of statins are needed in Asians than Westerners. When the same doses are administered, Asian patients show higher plasma levels of rosuvastatin and its metabolites than Westerners [55]. Therefore, Chinese people may tolerate rosuvastatin less at 20 mg/day than Westerners. This possibility partially confirmed when rosuvastatin was effective in POAF risk reduction when used in European people with the same dosage [26]. In addition, random allocation to 20 mg of daily rosuvastatin significantly reduced AF risks within the JUPITER (justification for the use of statins in primary prevention: an intervention trial evaluating rosuvastatin) trial, which was conducted in the United States on patients with underlying inflammation (CRP higher than 2 mg/L, without high low-density lipoprotein) [56]. However, 10 mg of daily rosuvastatin effectively prevented AF after catheter ablation in another Chinese trial, conducted on heart failure (HF) patients [57]. In any case, well-designed RCTs are needed to find the most effective treatment protocol of statins in multi-national patients after cardiac surgery.

Another possible explanation for the conflicting results of the STICS trial is that previous trials included only patients who were not statin users before the study. However, in the STICS trial, 653 of the 1922 patients (34%), including the placebo group, had used statins before recruitment [38]. Interestingly, another large trial that failed to show statin efficacy in POAF prevention included as high as 67% of statin-using patients [29]. However, we need more evidence to show whether statin therapy has a different effect between statin-naive patients and pre-study statin users [38]. After all, as discussed before, the resumption time of pre-study statin regimen can be a determinative factor in the results of such studies. However, a pre-specified subgroup analysis of the STICS trial failed to show any significant association between previous statin use and rosuvastatin efficacy in POAF prevention [25].

A clinical study comparing the effect of atorvastatin with rosuvastatin on POAF concluded that rosuvastatin or atorvastatin before surgery has a similar effect on preventing POAF [58]. It is notable that their study was a small study without any control group.

Concerning other statins, a large meta-analysis indicated that atorvastatin was more effective than pravastatin in reducing the rate of AF and using pravastatin failed to show protective effects (odds ratio (OR) 1.03, 95% confidence interval (CI) 0.77–1.37) [59]. However, this meta-analysis included all RCTs evaluating statin effectiveness in all types of AF, including POAF. This different effect of atorvastatin and pravastatin could be due to their different chemical properties. The higher lipophilicity of atorvastatin could have led to a higher affinity for the cardiac cellular membrane. As a result, more penetration to the cardiac muscle cells occurs. Therefore, it is more effective in reducing AF than a hydrophilic statin such as pravastatin and rosuvastatin [60,61].

### 4.3. Dose Response

It appears that statins exert their anti-arrhythmic properties due to their pleiotropic effect [39], which is dose-dependent. Very low doses of statins may lack pleiotropic effect and higher doses show higher pleiotropic effect [62]. Therefore, it is expected that higher statin dosage would be associated with more POAF reduction. However, the pleiotropic effect is not the only anti-arrhythmic mechanism of statins. Clinical studies led to conflicting or even surprising results. For instance, a meta-analysis of 20 RCTs concluded that statins effectively prevented AF and this AF risk reduction was dose-dependent [59]. Surprisingly, a subgroup analysis of RCTs showed that statin therapy was more effective in lower doses, particularly in the dose range of 10–40 mg/day of atorvastatin (OR 0.29, 95% CI 0.19–0.45) [59]. It is notable that the focus of this meta-analysis was not on POAF and included studies evaluating primary or secondary prevention from AF. However, the least statin dose, which has been used effectively in RCTs at new-onset POAF reduction, was 20 mg/d of atorvastatin (Table 1). As well, there are studies indicating that anti-inflammatory effect of statins is not dose-dependent [63], despite their pleiotropic effect. Additionally, other facts should be considered before discussing the results of the mentioned meta-analyses. First, the results on high-dose atorvastatin are inconsistent. Second, explanations for such conflicting results could be the following possibilities. The selection of high-dose statins may reflect the more severe conditions or more susceptibility of patients to AF in the study population. In these patients, inflammation has a less important role in AF occurrence, and the failure of high-dose statins in reducing POAF risk may be due to these severe conditions. As well, the usage of high-dose statins greatly increases the risk of drug interactions. As a result, it can reduce tolerability in the study population and may increase patient dropout rate. Therefore, it can affect the study results.

However, the results of some studies do not agree with our assumption about dose-response pattern of statins. Two studies, which compared high-dose with medium- or low-dose atorvastatin, showed no considerable differences in reducing the risk of POAF [28,46]. Similarly, another meta-analysis, which included eight RCTs evaluating the efficacy of statins on POAF occurrence, concluded that the dose of statins was not associated with risk reduction [64].

On the other hand, the results of some studies indicated that higher doses of statins cause better results. For instance, a retrospective study on patients who had CABG or valve surgery showed that the patients who were treated with simvastatin >20 mg daily had 36% lower odds of POAF (OR 0.64, 95% CI 0.43 to 0.6; *p* = 0.03) in comparison to those receiving lower dosages. Indeed, statin therapy with higher doses reduces the occurrence of POAF more efficiently [65].

After all, the current data fail to support more effectiveness of higher doses of statins. However, the majority of current data originate from non-placebo-controlled trials or subgroup analysis of few studies. Therefore, there is a need for well-designed studies to determine the optimum dose of statins for POAF prevention.

### 4.4. Effectiveness Associated with Surgery Type

Most of the trials confirming statin efficacy in POAF reduction were done on CABG and heart valve surgeries patients, isolated or combined (Table 1). However, some trials show statin efficacy also in other types of surgery including catheter ablation [57] or even in non-cardiac surgical procedures in high-risk patients [32].

Some meta-analyses have compared statin efficacy in POAF reduction between CABG and combined or isolated heart valve surgery. The results on statin efficacy in post-CABG AF reduction are consistent and show that statins are more effective in CABG patients [38,39,42]. CABG is a major surgery with more intensive inflammatory conditions. Therefore, the association between statin efficacy and post-CABG AF may be due to the association between inflammation, CABG, and AF.

### 4.5. Safety

There are several trials with statins without reporting any major side effect (Table 1 and Table 2). However, a recent large RCT, the STICS trial, showed that AKI occurred more frequently in the statin group [25]. The STICS trial was performed on Chinese patients who had cardiac surgery and were randomized to receive rosuvastatin 20 mg daily or placebo. This finding is in contrast to other trials. Billings et al. failed to show any relation between high-dose statin therapy and AKI [29], which was confirmed by two recent meta-analyses [38,53]. The only meta-analysis that confirmed the association between statin therapy and post-operative AKI in patients without renal dysfunction was greatly influenced by the STICS trial [41].

Anyway, the results of the STICS trial can be explained by the lesser tolerability of Chinese patients to high doses of rosuvastatin. There are some trials showing that the plasma levels of rosuvastatin and its metabolites were significantly higher in Asian, especially Chinese, populations [55,66,67,68]. As well, statins are metabolized with cytochrome p450, component CYP3A4, which can be a great potential source of drug interactions. High doses of statins, such as used in the STICS trial, greatly increase the risk of drug interactions leading to a significant increase in the serum levels of statins. As a result, the elevated levels of statins increase the risk and rate of side effects such as rhabdomyolysis. As is well known, rhabdomyolysis can lead to AKI [69]. Therefore, clinicians should be aware of such a drug interaction to prevent this potential side effect.

### 4.6. Other Benefits

Xia et al. showed that 500 stable coronary artery disease (CAD) patients, who underwent non-cardiac surgery, benefited from peri-operative atorvastatin reload. The statin group showed a lower rate of POAF and a lower incidence of major adverse cardiac events 30 days following surgery [32]. Moreover, a meta-analysis showed that pre-operative statin therapy was related to reduced stroke risk [44]. However, two other meta-analyses failed to find any association between statin therapy before surgery and reduced myocardial infarction (MI) rate [38,53].

Reduction in AF duration was documented in a trial that was carried out on patients undergoing isolated heart valve surgery and receiving 40 mg of atorvastatin pre-operatively [30]. In addition, several RCTs and meta-analyses showed that the statin group had a shorter length of ICU or hospital stay [32,34,36,38,40,42,63].

## 5. Conclusions

Statins, especially atorvastatin, in a special dose range, appear to be an effective option for POAF primary prevention, especially in patients who had CABG, which is an intensive heart surgery procedure associated with inflammation in the heart. However, there are few large studies indicating no beneficial effect of statins, especially rosuvastatin. One large clinical trial reported a higher risk of AKI following high-dose rosuvastatin in a Chinese population. In this study, however, rosuvastatin reduced the level of CRP but could not reduce the rate of POAF. Although several RCTs and meta-analyses have evaluated the efficacy of statins in POAF prevention, further studies are needed to find the most effective statin regimen for POAF prevention with the least safety considerations and the highest health benefits.

## Figures and Tables

**Table 1:** Randomized clinical trials evaluating the effect of statins on the prevention of post-operative atrial fibrillation.

Study Design	N of Patients	Surgery	Drug	Dose & Rout of Administration	Duration	Control	Results	Rate of Post-Operative Atrial Fibrillation	Inf. Markers	Safety	Reference
**R PC clinical trial**	1922	elective CABG, Aortic valve surgery or both	rosuvastatin	20 mg/d	For up to 8 days before surgery until 5 day after surgery	placebo	No significant differences between groups (OR 1.04 95% CI 0.84–1.3 p 0.72)	21.1% in rosuvastatin group and 20.5% in placebo group	CRP levels were lower significantly	More postoperative acute kidney injury rate and no beneficial effect	[25]
**R clinical trial**	212	Elective on-pump CABG	atorvastatin	80 mg/d For 7 days before surgery, stopped the evening before, resumed 4 h later with 40 mg/d		Atorvastatin 40 mg	A trend toward a decrease in POAF was observed with high-dose statin but did not reach to significance	23.6% in atorvastatin 40 mg group and 15.8% in atorvastatin 80 mg group	Mean values of CRP and IL6 were not different between 2 doses		[28]
**R DB PC clinical trial**	199 statin-naïve 416 statin user	cardiac surgery	atorvastatin	80 mg/d At the day before surgery or the morning of surgery followed by 40 mg/d		placebo	POAF increased in statin-native patients and statin-native patients with chronic kidney disease vs. control, but in all patients, statin did not affect POAF (RR, 1.11 0.90, 1.38 P 0.38)	37.3% in atorvastatin group And 33.6% in placebo group			[29]
**R DB PC** **Clinical trial**	58	On-pump isolated valve surgery	Atorvastatin	40 mg/d	From 3 days before surgery until 5 POD	placebo	Significant lower rate of POAF (OR 0.122 95%CI 0.027–0.548 p 0.006)	21% in atorvastatin group and 45% in placebo group	Lower WBC count in statin group	Lower AF duration	[30]
**P R clinical trial**	60	Elective isolated CABG	Atorvastatin	40 mg/d	from 6 h PO and continued	Non-statin	Significant lower rate of POAF (OR 0.512 95% CI 0.005– 0.517 p 0.012)	16.7% in statin group and 43.3% in placebo group			[31]
**R PC clinical trial**	500 stable CAD patients	Non-cardiac surgery	Atorvastatin reload peri-operatively			Placebo	Significant lower rate of POAF (p 0.0003)	6.8% in statin group and 17% in placebo group		Lower rate of 30-days incidence of major adverse cardiac events	[32]
**R DB PC** **Clinical trial**	60	Isolated first-time CABG	Atorvastatin	40 mg/d	From 14 days before surgery afterwards	Placebo	Significant lower rate of POAF compared to placebo (3.3% vs. 23% p 0.02)	3.3% in atorvastatin group and 23% in placebo group	CRP levels were significantly lower pre- and post-operatively in statin group		[33]
**R clinical trial**	90 (30 in each group)	Elective CABG	Atorvastatin	20 mg/d	From 3 weeks before surgery afterwards	Non-statin and non-corticosteroid	Significant lower rate of POAF	13.8% in atorvastatin group, 10.3% in MP group and 39.3 % in placebo group	PO IL-6 levels were lower compared to control	Increased PO cardiac index and reduced ICU stay	[34]
**R single-blind** **Clinical trial**	104	CABG or Aortic valve replacement	Atorvastatin	80 mg/d	From 7 days before surgery	Atorvastatin 10 mg/d	A non-significant reduction in POAF by high-dose statin vs. low-dose statin	29% in high dose group vs. 36% p 0.43			[35]
**R PC clinical trial**	100	Elective on-pump CABG	atorvastatin	20 mg/d	From 7 days before surgery	placebo	A significant reduction in POAF by statin vs. placebo (OR = 0.235, *p* = 0.007)	(18% in statin group vs. 41% in placebo group p 0.017)	PO peak CRP levels were lower in statin group		[27]
**R PC clinical trial**	200	Elective CABG	Rosuvastatin	20 mg/d	From 7 days before surgery	placebo	A significant reduction in POAF by statin vs. placebo (OR 0.46 95%CI 0.22–0.94 P 0.03)	18% in rosuvastatin group and 35% in placebo group	Lower CRP increasement above than a AF-predictor levels		[26]
**R fully-blinded PC clinical trial**	200	On-pump CABG or valve surgery	atorvastatin	40 mg/d	From 7 days before surgery	placebo	A significant reduction in POAF by statin vs. placebo (OR 0.39 95%CI 0.18-0.85 P 0.017)	(35% versus 57%, *p* = 0.003)	Peak CRP levels were lower in patients without AF (*p* = 0.01), irrespective of statin use	Similar major adverse cardiac and cerebrovascular events at 30 days	[36]

**Abbreviations**: CABG (Coronary artery bypass grafting), CRP (c-reactive protein), N (number), POAF (Postoperative atrial fibrillation), RR (risk ratio), OR (odds ratio)

**Table 2:** Meta-analyses evaluating the effect of statins on the prevention of post-operative atrial fibrillation.

N and Design of Included Studies Evaluating POAF	N of Sample	Was Effective POAF Risk Reduction?	In Which Surgery Type Are Statins More Effective?	Which Statin Is More Effective	Other Benefits or Any Hazards	Changes in Inflammatory Markers	Reference
**18 RCT**	3995	Yes RR 0.69 95%CI 0.56–0.86 p 0.001	Only in CABG	NA	Not associated with reduced or increased risk of AKI or MI, but there was an increased trend of higher AKI in patients with valve surgery	Significant decrease in inflammatory response	[37]
**20 RCT**	4338	Yes RR 0.50 *p* = 0.0004	Isolated CABG, not effective in combined surgeries	Ator	Not effective in post-operative AKI or MI	NA	[38]
**12 RCT**	1116	Yes, OR 0.50 95%CI 0.41–0.61 *p* = 0.00001	CABG	NA	NA	NA	[39]
**15 Clinical trials**	9369	Yes OR 0.481 95% CI 0.345–0.672 *p* = 0.00	Only studies evaluating post-CABG AF included	NA	Significant decrease in cerebral circulation disorders	Significant decrease in inflammatory markers	[40]
**3 RCTs with low-risk of bias**	2637	No	NA	NA	26% increase in AKI. No association between statin therapy and risk of MI	NA	[41]
**12 RCT**	2980	Yes 0R 0.42 95%CI 0.27–0.66 p 0.0001	Only studies evaluating post-CABG AF included	Only Ator not Rosu	NA	NA	[42]
**All related published study**	NA	Yes Homogenous OR 0.37 95%CI 0.28–0.51 *p* < 0.0001	NA	NA	NA	NA	[43]
**12 (3 meta-analysis, 5 RCTs and 4 retrospective studies)**	NA	Pre-operative statin use, yes	NA	NA	Significant reduction in risk of stroke	Reduced levels of inflammatory markers	[44]
**12 RCTs**	1765	Pre-operative statin use, yes OR 0.54 95%CI 0.43–0.67 *p* < 0.01	NA	NA	No reduction in MI or renal failure No major or minor side effect of statins	NA	[45]
**11 RCTs**	1105	Pre-operative statin use, yes OR 0.41 95%CI 0.31-0.54	NA	NA	NA	NA	[46]
**12 RCTs**	NA	Yes RR 0.50 95%CI 0.35–0.73	NA	NA	NA	NA	[47]
**8 RCTs**	1156	Yes, it reduce the new-onset POAF OR 0.44 95%CI 0.29–0.68 *p* < 0.0002	NA	Ator	NA	Reduced	[48]
**26: RCTs and** **observational studies**	28,772	In pre-operative statin use, yes OR 0.72 95%CI 0.59–0.87	post-CABG studies included	NA	No significant changes in odds for renal failure	NA	[45]
**RCTs**	NA	Yes RR 0.7 0.54–0.91	NA	Rosu	NA	It was effective especially in patients with higher CRP levels	[49]

**Abbreviations** AF (atrial fibrillation), AKI (Acute kidney injury), CABG (Coronary artery bypass grafting), CRP (c-reactive protein), N (number), POAF (Postoperative atrial fibrillation), NA (not available), RR (risk ratio), OR (odds ratio).
